# A short review article on conjugated polymers

**DOI:** 10.1007/s10965-023-03451-w

**Published:** 2023-02-23

**Authors:** Akhtar Hussain Malik, Faiza Habib, Mohsin Jahan Qazi, Mohd Azhardin Ganayee, Zubair Ahmad, Mudasir A Yatoo

**Affiliations:** 1 Government Degree College Sopore, Department of Chemistry, 193201 Jammu and Kashmir, India; 2 Department of Chemistry, University College London, Gordon St, London, WC1H 0AJ UK; 3 Meining School of Biomedical Engineering, Cornell University, Ithaca, NY 14853 USA; 4 Department of Chemistry, Indian Institute of Technology Madras, 600036 Chennai, India; 5 School of Chemical Engineering, Yengnam University, 38541 Gyeongbuk, Republic of Korea; 6 Department of Materials, Imperial College London, Exhibition Road, London, SW7 2AZ UK

**Keywords:** CPEs, Sensors, PET, RET, FRET, Quencher, Coupling reactions

## Abstract

This article provides a brief review of conjugated polymers and the various typical polymerization reactions exploited by the community to synthesise different conjugated polyelectrolytes with varied conjugated backbone systems. We further discuss with detailed emphasises the mechanism involved such as photo-induced electron transfer, resonance energy transfer, and intra-molecular charge transfer in the detection or sensing of various analytes. Owing to their excellent photo-physical properties, facile synthesis, ease of functionalization, good biocompatibility, optical stability, high quantum yield, and strong fluorescence emission. Conjugated polymers have been explored for wide applications such as chemical and biological sensors, drug delivery and drug screening, cancer therapeutics and imaging. As such we believe it will be a timely review article for the community.

## General introduction

Conjugated polymers (CPs) are organic macromolecules having alternating σ and π bonds along their backbone. The delocalized π-electron cloud over the backbone chain is responsible for the attractive optical and electrochemical properties of CPs. The conducting nature of CPs was first discovered [1] and developed by Alan MacDiarmid, Hideki Shirakawa and Alan J. Heeger in 1977 which led them to win the chemistry Nobel Prize in 2000. CPs has attracted significant attention over the last three decades because of their technologically promising future. These CPs have been explored for a variety of applications, including solar cells [2], light emitting diode (LEDs) [3, 4], organic electrochemical transistors (OECTs) [5], energy storage systems [6–8], field-effect transistors (FETs) [9, 10], and chemo- and biosensors [11, 12].

**Fig. 1:**
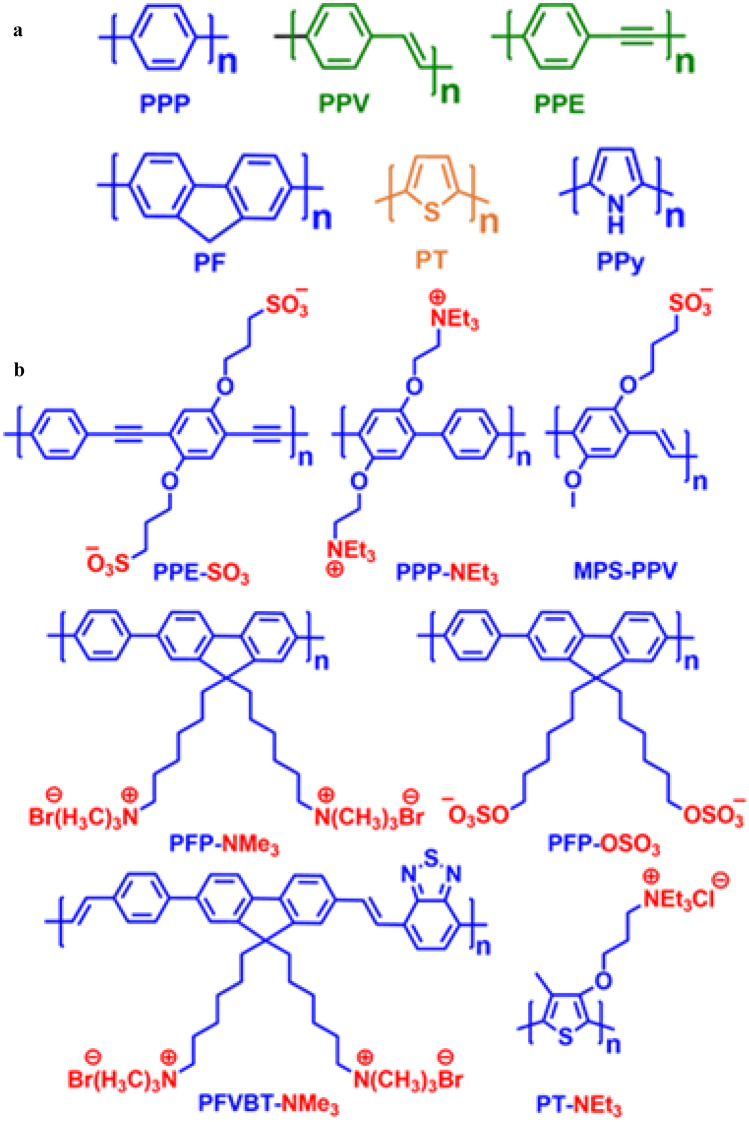
Structures of some (**a**) common conjugated polymer backbones (**b**) selected examples of conjugated polyelectrolytes

CPs can be acquired with variable backbone chains, viz. poly (p-phenylene) (PPP), poly (p-phenylene vinylene) (PPV), poly (p-phenylene ethynylene) (PPE), polyfluorene (PF), polythiophene (PT), polypyrrole (PPy), etc. as shown in Fig. 1a. Conjugated polyelectrolytes (CPEs) are water-soluble conjugated polymers having pendant ionic functionalities such as sulfonate (SO_3_^−^), carboxylate (CO_2_^−^), phosphonate (PO_3_^2−^) and quaternary ammonium (NR_3_^+^) appended with the main conjugated chain that assists them to ionize in high dielectric media and forming stable electrostatic complexes with target analytes [13–16]. These materials combine the optoelectronic properties of neutral conjugated polymers (CPs) as well as the electrostatic behaviour of polyelectrolytes, thus, providing an exceptional platform in the field of sensors (chemical and biological) [12, 17–19] because of their capability to transform a binding/unbinding event into a measurable optical or electrochemical response, excellent biocompatibility, low cytotoxicity, strong emission brightness and resistance to photo-bleaching. Some examples of CPEs are presented in Fig. 1b above.

### Synthesis of conjugated polymers

During the past few decades, a large number of CPEs have been designed and synthesised using the same conjugated backbone structure. Based on the structure of backbone moiety CPs can be classified into various types, namely poly(fluorene-co-phenylene) (PFP), poly(p-phenylenevinylene) (PPV), poly(p-phenyleneethynylene) (PPE), polydiacetylenes (PDA), and poly(thiophene) (PT). For the synthesis of various CPEs commonly employed reactions (Suzuki [20], Heck [21], and Sonogashira [22], Wessling reaction [23], photopolymerization reaction [24], and FeCl_3_ oxidative polymerization [25]) are shown in Table 1.

**Table 1:**
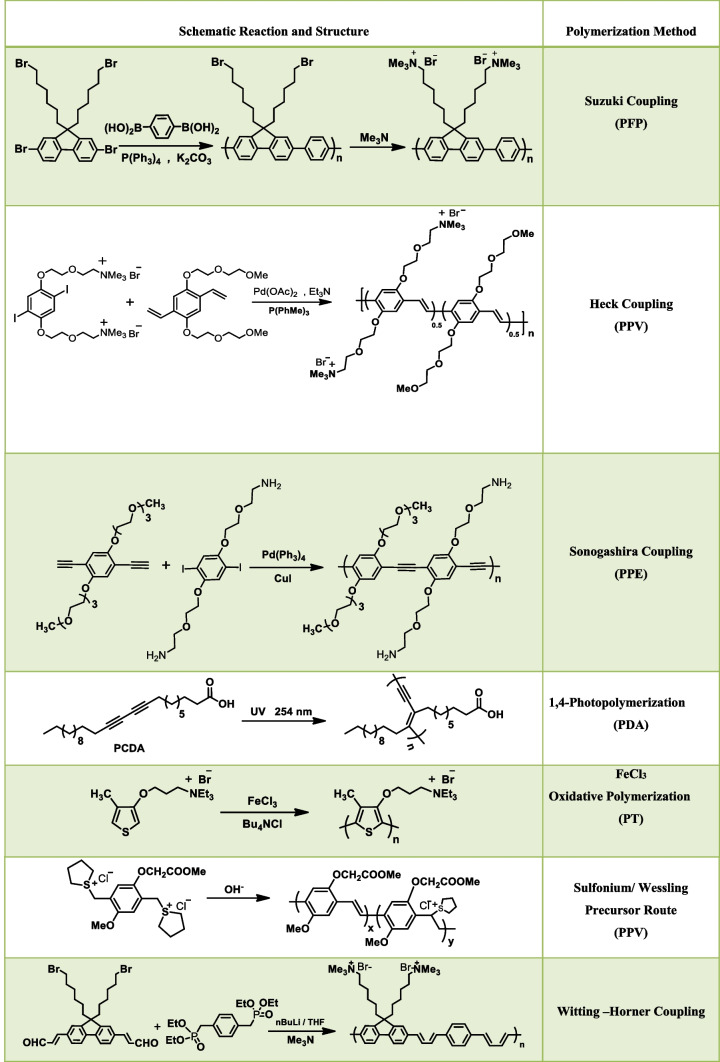
Common polymerization reactions for the synthesis of CPEs

### Amplified quenching effects

One of the interesting properties of conjugated polymers is the process of efficient fluorescence quenching at low quencher concentrations, which is referred to as amplified quenching [12, 26]. It was Swager and co-workers in 1995 who for the first time demonstrated this phenomenon [26, 27] of super quenching in polymers via the “molecular-wire effect” which is responsible for its superior sensitivity over small molecule indicators. This signal amplification is an outcome of the ability of the CP to generate exciton (bound electron–hole pair) which migrates efficiently along the conjugated backbone. Because of the efficient charge migration, a single quencher unit bound to the receptor site of the polymer will quench the fluorescence of almost the entire polymer chain leading to the amplified quenching response to a target analyte. This incredible feature of CPs validates its widespread application as chemo- and biosensors, since a very dilute concentration of the analyte is required.

#### Stern-volmer fluorescence quenching

1 $$\mathrm{X}^{\ast}+\mathrm{Q}\stackrel{\mathrm{kq}}{\to }\mathrm{X}+\mathrm{Q}$$

2 $$\mathrm{X}+\mathrm{Q}\overset{\mathrm{ka}}\rightleftharpoons\left[\mathrm{X},\mathrm{Q}\right]\xrightarrow{\mathrm{hv}}\left[\mathrm{X}^{\ast},\mathrm{Q}\right]\rightarrow \mathrm{X}+\mathrm{Q}$$

3 $${\mathrm{I}}_{0}/\mathrm{I}=1+{\mathrm{k}}_{\mathrm{sv}}[\mathrm{Q}]$$

From Eqs. (1) and (2), X* is an excited-state fluorophore, Q is a quencher molecule, kq is the bimolecular quenching rate constant, and ka is the association constant for the ground-state complex formation [X, Q].

**Fig. 2:**
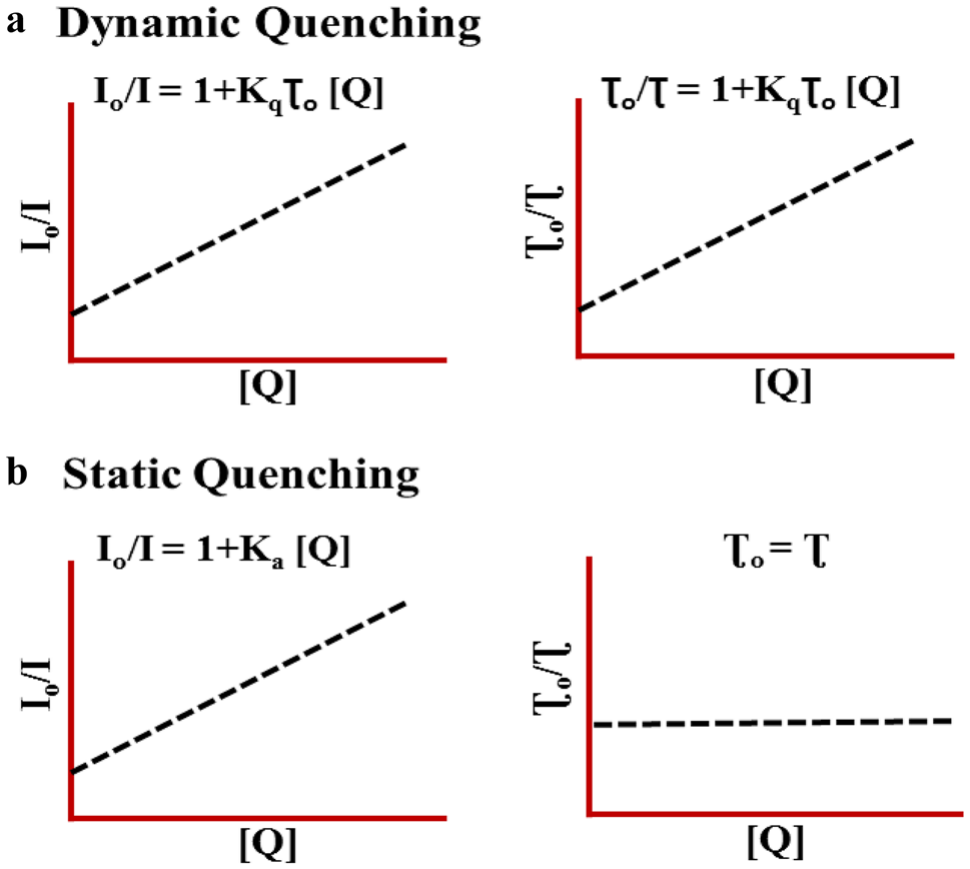
Plots showing the effect of quencher concentration on fluorescence emission and fluorescence lifetime in case of **a** Dynamic and **b** Static quenching

Equation (3) is known as the Stern-Vomer equation, where I_0_ is the fluorescence intensity in absence of a quencher, I is the fluorescence intensity in presence of a quencher, and K_sv_ is the Stern–Volmer quenching constant. Fluorescence quenching can take place by two different mechanisms i.e., dynamic (collisional) quenching and static (complex formation) quenching. Dynamic quenching (Eq. (1)) occurs when the excited-state fluorophore encounters the quencher molecule which can facilitate non-radiative transitions to the ground state and the fluorescence is quenched. Static quenching takes place by binding the quencher molecule to the fluorophore. The excited fluorophore generated is immediately and quantitatively quenched (Eq. (2)). In the case of dynamic quenching, K_sv_ = kqτ_0_, where τ_0_ is the fluorescence lifetime of X*. The lifetime of the fluorophore in this case is reduced in the presence of a quencher (Fig. 2a). On the other hand, K_sv_ = Ka, if quenching is dominated by the static mechanism and its lifetime remains unaffected in presence of a quencher (Fig. 2b). In both static and dynamic quenching, the Stern–Volmer plots of I_0_/I versus [Q] should be linear as per Eq. (3). However, in most cases, the Stern–Volmer plots show non-linearity (curved graph) which can be explained by various complex processes, such as variation in the association constant with quencher concentration, mixed dynamic and static quenching mechanism, and chromophore aggregation.

**Fig. 3:**
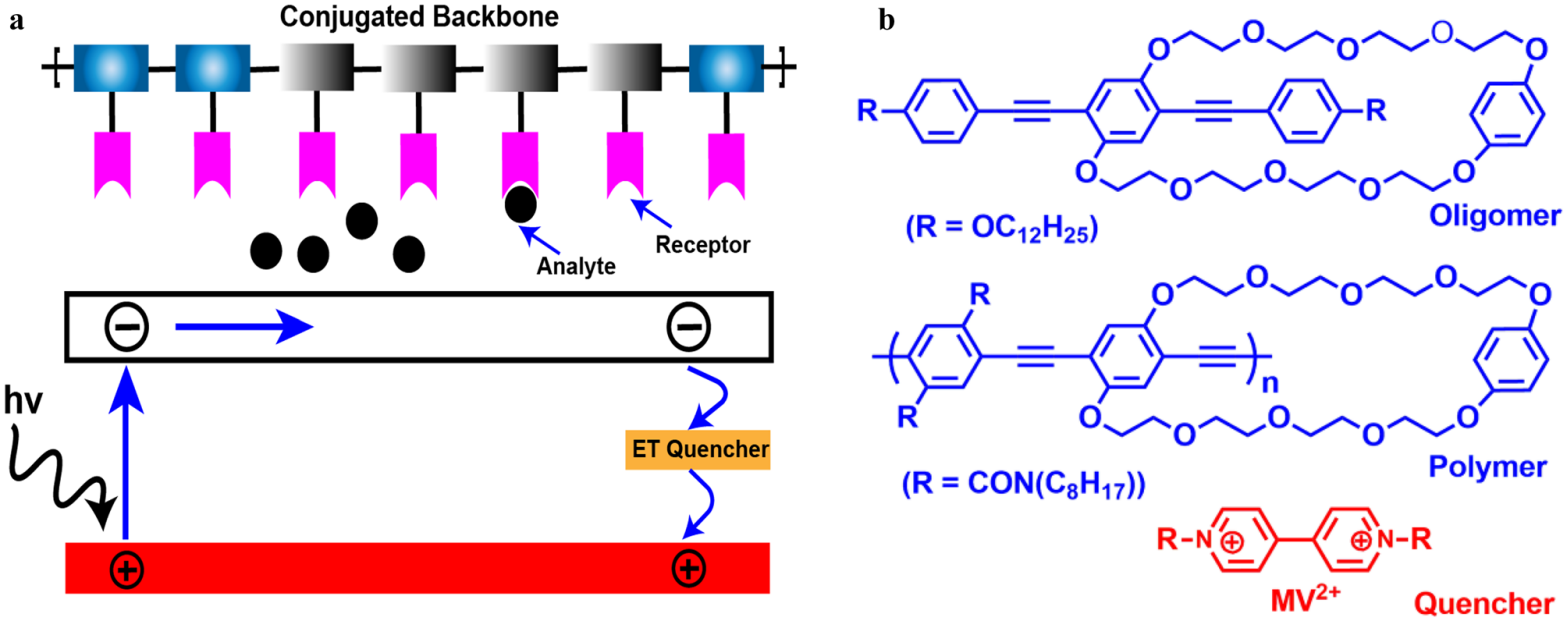
**a** Quenching mechanism of molecular wire effect in conjugated polymers **b** structure of oligomer, polymer, and quencher (MV^2+^) used as fluorescence chemosensor for the first time by Swager and co-workers

The proposed mechanism of this effect is efficient energy migration along the polymer backbone as discussed above. In 1998 Yang and Swager explored the above phenomena of signal amplification for the sensitive detection of nitro-explosives 2,4,6-trinitrotoluene (TNT) and 2,4-dinitrotoluene (DNT) [28, 29]. However, the super quenching effect in CPEs was first described by Whitten and co-workers in the study of the fluorescence quenching of MPS-PPV by MV^2+^ (Fig. 3b) [30]. The fluorescence of the MPS-PPV solution was efficiently quenched by MV^2+^, with a large K_sv_ value of 10^7^ M^−1^. This amplified quenching was attributed to the strong electrostatic interaction between the negatively charged polymer and MV^2+^ which bring them in close proximity resulting in disruption of exciton diffusion along the polymer backbone which leads to efficient fluorescence quenching (Fig. 3a). This study showed that polymer fluorescence is quenched much more efficiently than oligomers.

### Conjugated polymers used as chemo or biosensors

Over the past few decades, CPs have been extensively used as sensory materials for various chemical and biological species [11, 12, 17–19, 31–33] such as metals ions, anions, environmental pollutants, explosive materials, proteins, enzymes, etc. due to their remarkable sensitivity, high photoluminescence quantum yield, large extinction coefficients, photo- and thermal stability, etc. One of the important features of CPs is their capability to transform a binding/unbinding event into a measurable response (electrochemical or optical) which makes them a material of choice for sensing applications.

In general, CP-based fluorescent sensors can operate either in “turn-off” or “turn-on” modes. In the former, the polymer is fluorescent without a quencher, and its fluorescence is turned off upon the addition of the analyte whereas, in the latter, fluorescence is recovered once the quencher is added. Most of the CPs work on the principle of non-covalent interaction-based sensing mechanisms such as photo-induced electron transfer (PET), resonance energy transfer (RET), inner filter effect (IFE), and intramolecular charge transfer (ICT) to detect various analytes. In the next subsections, we will discuss the mechanisms in detail.

#### Photo-induced electron transfer

**Fig. 4:**
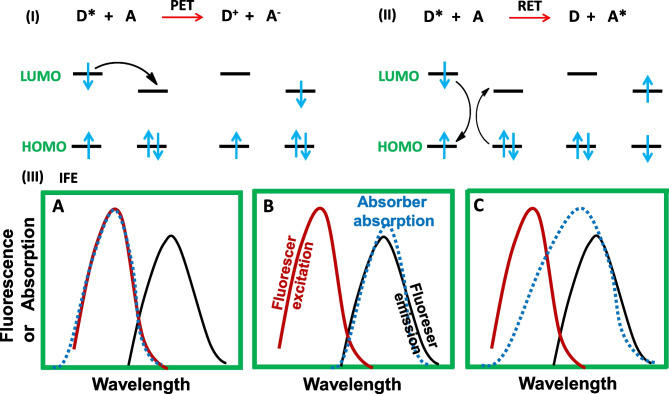
Elucidation of the sensing mechanism in conjugated polymers **i** PET and **ii** RET **iii** IFE: **A** absorption spectrum of absorber overlaps with the excitation of fluoreser **B** absorption spectrum of absorber overlaps with emission spectrum of fluoreser **C** both excitation and emission spectrum of fluorescer overlaps with absorption spectra of absorber

In a PET sensing system, complexation takes place between the excited electron donor (D*) and the electron acceptor species (**A**), here D* donates its electron to **A** producing a complex [**D**^**+**^**-A**^**−**^] (Fig. 4i) [34]. Here, the charge transfer (CT) complex relaxes to the ground state via non-radiative transition and the additional electron present on the acceptor can finally return to the electron donor. Thus, PET plays an important role in the process of fluorescence quenching.

#### Resonance energy transfer

In RET, acceptor **A** absorbs energy from the excited donor D* while relaxing to the ground state and getting excited to A* (Fig. 4ii). Here, in this case, the rate of energy transfer depends on the relative orientation of the donor and acceptor dipoles, the extent of overlapping of the emission donor and the absorption acceptor, and the distance between the donor and acceptor [12, 35]. Moreover, RET occurs due to the dipolar interaction between the excited donor and acceptor, thus it’s not sensitive to steric factors [36, 37].

#### Intramolecular charge transfer

In ICT, electron/charge transfer takes place from electron-rich moiety to electron-deficient located in the same molecule. ICT primarily takes place in the photo-excited state of the molecule which facilitates electron transfer from one part of the molecule to another in the excited state. Generally, ICT occurs in those molecules in which donor–acceptor moieties are linked with each other via a π-electron cloud. However, charge transfer can also take place through space but the donor and acceptor must have a favourable orientation.

#### Inner filter effect (IFE)

IFE was observed by stokes [38]. It’s an important energy conversion model (non-irradiation), resulting from absorption of the excitation or emission light by the absorber [39]. Earlier IFE was considered a problem in fluorescence measurements especially when the analyte with absorbing nature was titrated [40]. In recent years, IFE has emerged as a superior technique in the field of sensing. Two optical units (absorber and fluorescer) are required for the establishment of an IFE based sensing system and for excellent results following points are to be considered. (a) There should be sufficient overlap between the absorption spectrum of absorber and the excitation/emission spectrum of fluorescer. (b) For quantitative analysis of analyte, the absorption spectra of absorber should be sensitive to the analyte concentration. (c) There should be no influence of external agents on the fluorescence and absorption of fluorescer and absorber respectively. (d) There should not be any effect of analyte on the fluorescence emission of fluorescer. (e) The absorber and fluorescer should exist as charge repulsion pairs in order to avoid fluorescence quenching of the fluorescer. Thus IFE-based system provides a simple and flexible method in the field of analytical detection.

### Detection of explosives

**Fig. 5:**
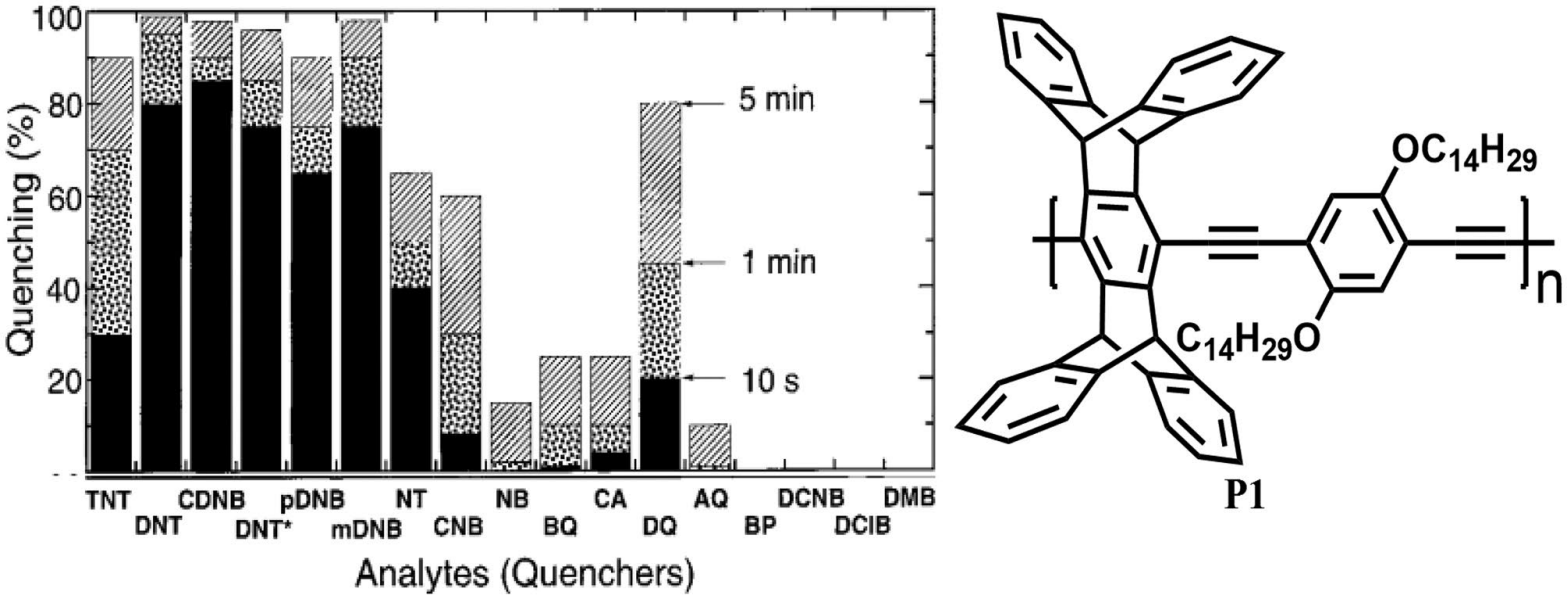
Quenching response of CP P1 (pentiptycene derivative) towards vapours of different nitroaromatic analytes including TNT. (The figure is reproduced with permission from Yang and Swager [28] Copyright © 1998, American Chemical Society)

One of the most successful applications of CP sensors is in the detection of electron-deficient nitro-aromatic explosives, especially 2,4,6-trinitrotoluene (TNT), trinitrobenzene (TNB), Picric acid (PA) which are of great current interest in both national security and environmental protection because they are not only explosives but also recognized as toxic pollutants [41]. In 1998, Yang and Swager [28] used a fluorescence quenching transduction mechanism along with the amplifying nature of CPs to design **P1** (Fig. 5) as a highly sensitive material to detect TNT vapour. An important feature of this design is the rigid pentiptycene group, which prevents the chains of polymer from aggregating and displaying solution-like optical properties in thin films, therefore, preventing the quenching interaction that can hinder the exciton migration. Polymer **P1** was able to detect TNT at a concentration of 10 ppb after a few seconds of exposure, thus exhibiting extraordinarily high sensitivity because of energy migration through polymer films.

**Fig. 6:**
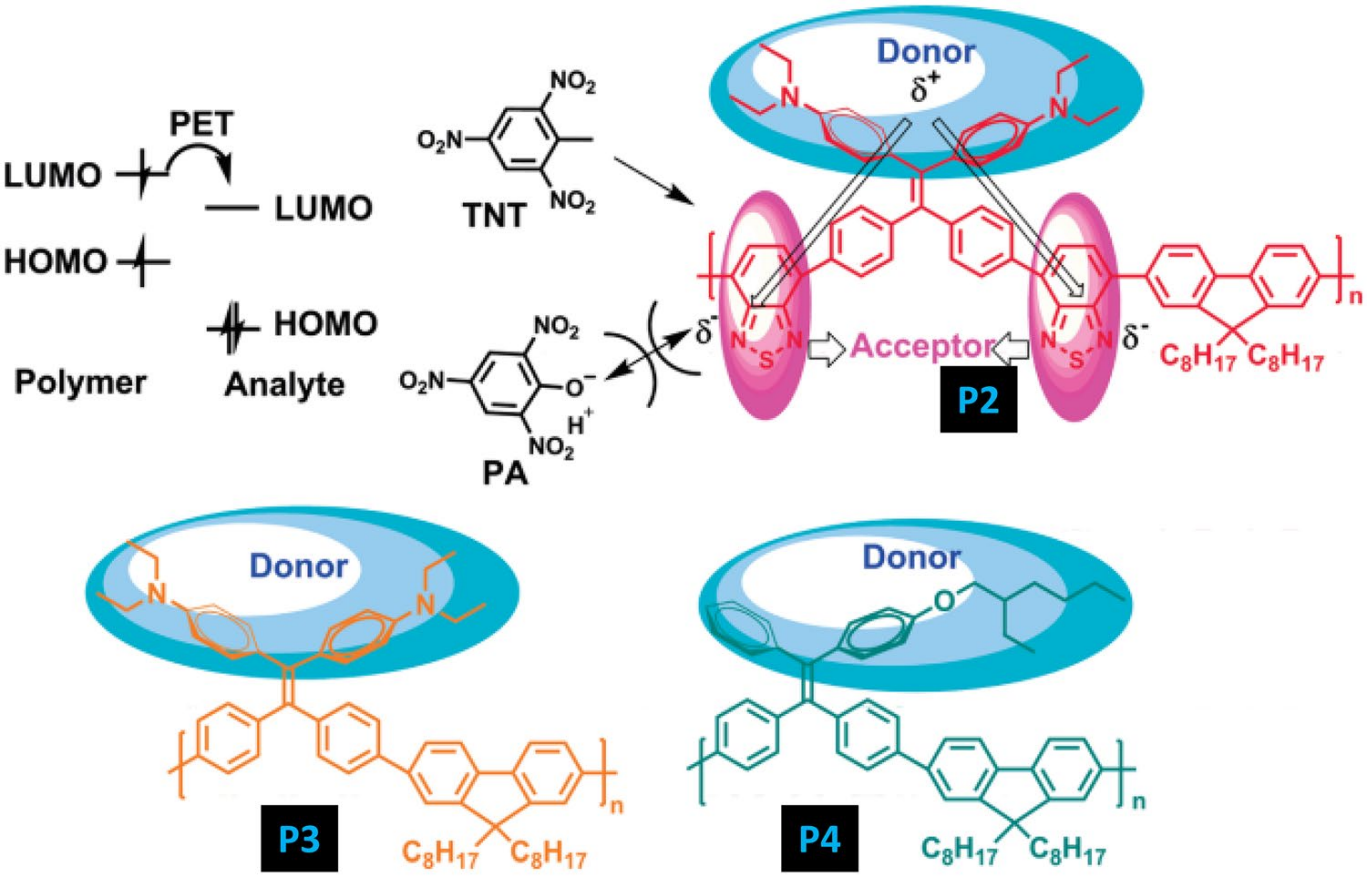
CPs P1, P2 & P3 has been explored for the selective and sensitive detection of PA and TNT in aqueous media. (The figure is reproduced with permission from Xu et al. [42] Copyright © 2011, American Chemical Society)

It has been observed mostly that PA exhibits higher quenching efficiencies than TNT because of its lower LUMO energy level. Therefore, detecting TNT in presence of PA in an aqueous solution is challenging. In this regard, Xu et al. [42] in 2011, designed donor–acceptor **(P2)** and donor-only (**P3** and **P4**) AIE active conjugated polymers (Fig. 6) for the selective detection and discrimination of TNT and PA in aqueous solution. In **P2** the LUMO is mainly localized on the acceptor unit of the CP i.e., 2,1,3-benzothiadiazole (BT). Since PA can easily form a negatively charged anion in an aqueous solution, an electrostatic repulsive interaction between the BT unit and PA will disrupt the efficient electron transfer from the LUMO of **P2** to PA which will not occur in the case of TNT. Therefore, **P2** can selectively detect TNT instead of PA in an aqueous solution. In the case of polymer **P3** and **P4** having only donor groups behave similarly to most CP sensors i.e., having greater selectivity toward PA rather than TNT. Furthermore, these polymers were spin-coated and then exposed to the vapours of TNT and PA. For **P1** film, the *K*_sv_ of TNT (1.2 × 10^5^ M^−1^) is almost 100 times more than that of PA (1.8 × 10^3^ M^−1^) and the limit of detection for TNT is about 23 ppb, while the emission of the **P4** film was selectively quenched by PA with *K*_sv_ constant of 2.8 × 10^4^ M^−1^ and the detection limit of 2 ppb was observed. The fluorescence quenching response follows the order of DNT > TNT > PA, which can be explained on the basis of the differential vapour pressures of these analytes. Higher quenching for DNT can be attributed to its higher vapour pressure and vice versa in the case of PA.

**Fig. 7:**
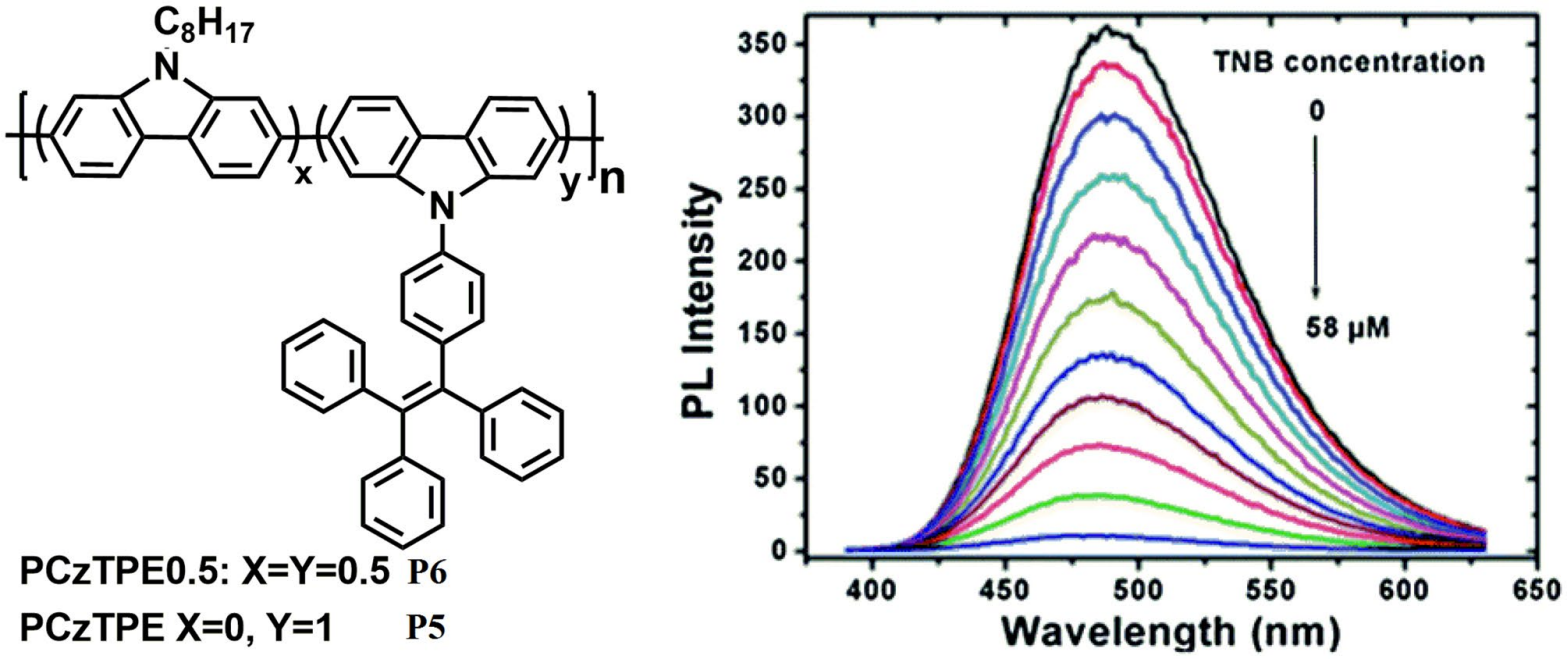
AIE active CPs P5 and P6 showed amplified quenching in presence of TNB. (The figure is reproduced with permission from Dong et al. [43] Copyright © 2014, Royal Society of Chemistry)

In 2014, Dong et al. [43] synthesized two poly (3,6-carbazole) **P5** and **P6** (Fig. 7) with AIE-active tetraphenylethylene (TPE) on their side chains via nickel-catalysed Yamamoto coupling under microwave heating. The resulting polymers combine the electron-deficient behaviour of the polycarbazole and the AIE-active nature of TPE and display distinct AIE properties. **P6** shows amplified fluorescence quenching response upon adding trinitrobenzene (TNB) in (1: 9, v/v) THF-water mixtures with a quenching constant of 1.26 × 10^6^ M^−1^. This enhanced quenching is ascribed to the increased number of quenching sites due to the twisted 3D topology of the polymer in the nanoaggregates that can interact with TNB molecules. The mechanism of quenching was deduced to be excited electron transfer from the LUMO of the polymer to the LUMO of TNB.

Furthermore, for practical applications, Whatman filter paper strips were cut and dip-coated into polymer solutions followed by drying in air. Both the polymers show TNB-induced fluorescence quenching in solution as well as in the vapour phase thus demonstrating the potential in solid state sensors for nitroaromatic explosives.

**Fig. 8:**
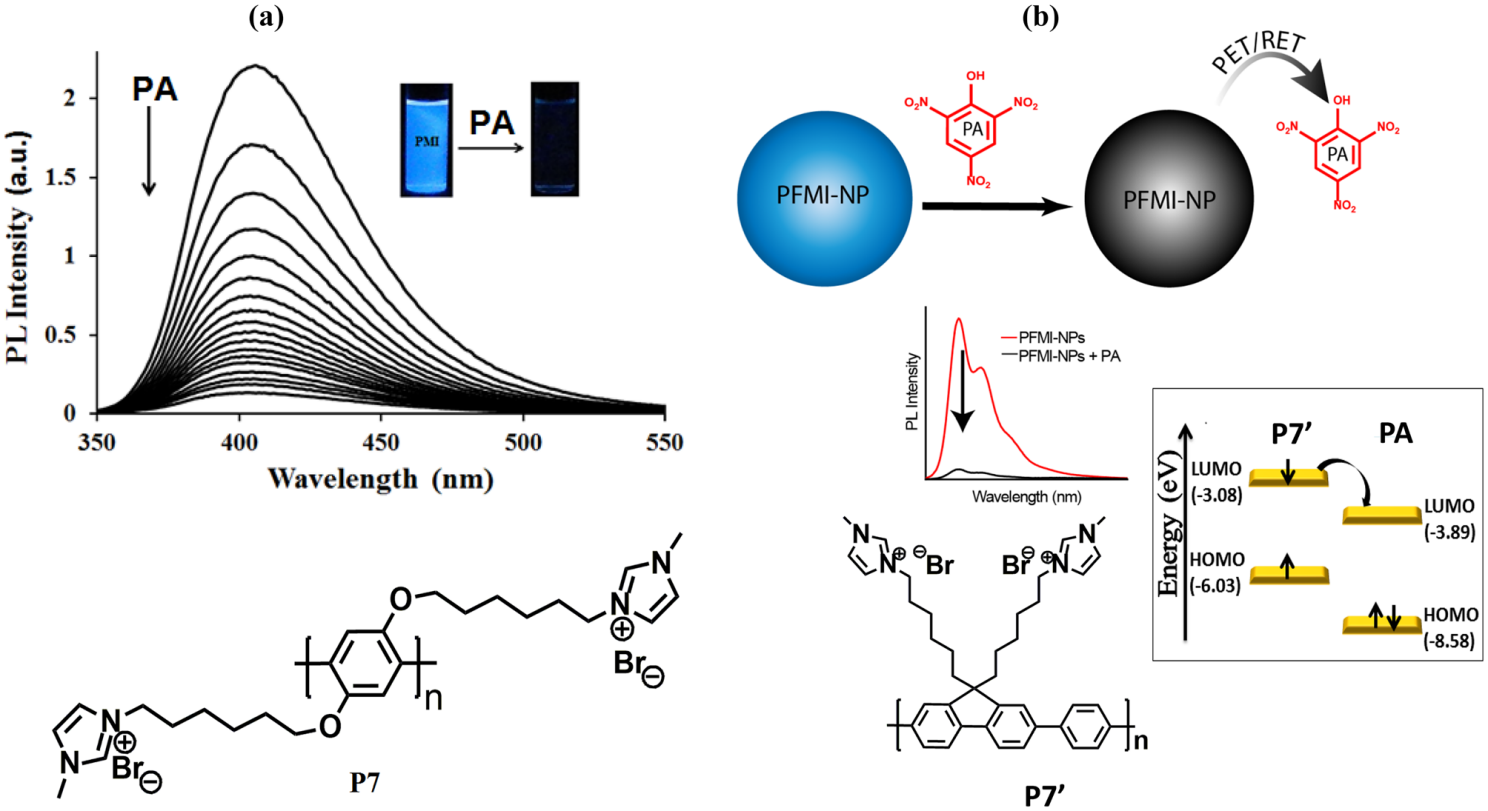
**a** Structure of P7 used for PA detection and **b** P7ˈconverting to nanoparticles for the detection of PA on multiple platforms. (The figure is reproduced with permission from Hussain et al. [44] and Malik et al. [45] Copyright © 2015, Royal Society of Chemistry & Copyright © 2015, American Chemical Society respectively)

In 2015*,* Hussain et al. [44] developed a new strategy of introducing an ionic functionality onto the side chain of the polymer as a receptor for the specific interaction of CP with the target analyte. The methyl imidazolium group was introduced into the precursor polymer system to form a cationic polymer **P7** (Fig. 8a). Since PA exists as a negative entity in an aqueous solution and the receptor attached to the CP is positively charged so there is a strong electrostatic attraction which brings the PA in close vicinity resulting in efficient charge transfer or energy transfer from the conjugated polymer to the analyte and ultimately amplified fluorescence quenching is obtained. Here, in this case, the highest quenching constant *K*_sv_ of 1 × 10^7^ M^−1^ and a detection limit of 128 ppt were obtained which confirms the method to be highly sensitive towards PA. Furthermore, paper strips and polymer-doped chitosan films were used for the contact mode detection of PA which confirms the method to be practicable. In the same year, Malik et al. [45] developed conjugated polymer nanoparticle (CPN) via re-precipitation technique based on multiple platforms (i.e.100% water, disposable film and as devices in vapour phase) for the sensing of PA (Fig. 8b). The highest quenching constant (K_sv_) of 1.12 × 10^8^ M^−1^ and a very low detection limit of 7.07 ppt was obtained. The reason behind this ultra-sensitivity was electrostatic interaction, photo induced electron transfer (PET) and/or possible resonance energy transfer (RET) between the quencher molecule and the CPN system. The origin of the remarkable selectivity and the exceptional quenching efficiency by PA can be explained via the electrostatic interaction between PA and P7***ˈ***. To investigate the possibility of electron transfer process in the quenching mechanism, HOMO and LUMO levels of P7**ˈ** was obtained and it was found that there is a possibility of excited state electron transfer from LUMO of P7***ˈ*** (-3.08 eV) to the LUMO of PA (-3.89 eV) resulting in the quenching process.

### Detection of anions

**Fig. 9:**
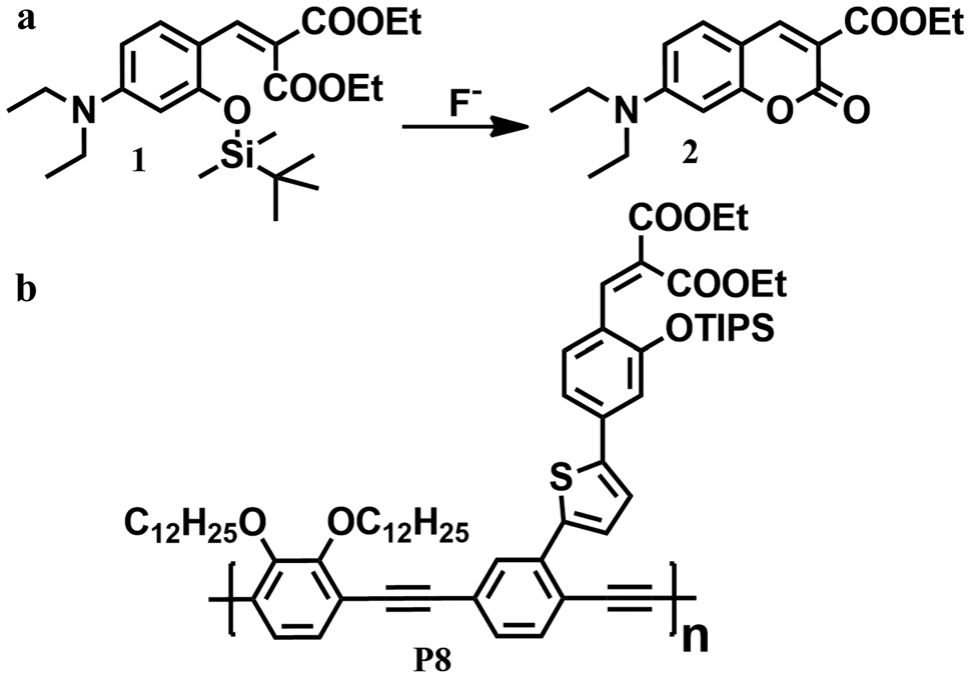
**a** Fluoride triggered cleavage of Si–O bond. **b** CP P8 for the detection of fluoride ion via exciton migration

It is known that selective detection of fluoride anion can be achieved by exploring the unique reactivity of fluoride towards silicon. As shown in Fig. 9a fluoride triggered the removal of silyl from compound **1** which results in the formation of a highly emissive compound **2** (coumarin fluorophore). Kim et al. [46] in 2003 designed polymer **P8** (Fig. 9b) and applied the above strategy for the amplified detection of fluoride by exploiting the exciton-transporting property of CPs. As anticipated, fluoride-induced formation of coumarin residues along the polymer chain acts as local band gap traps for the migrating excitons and thus gives an enhanced signal response up to 100-fold compared to single molecule-based detection.

**Fig. 10:**
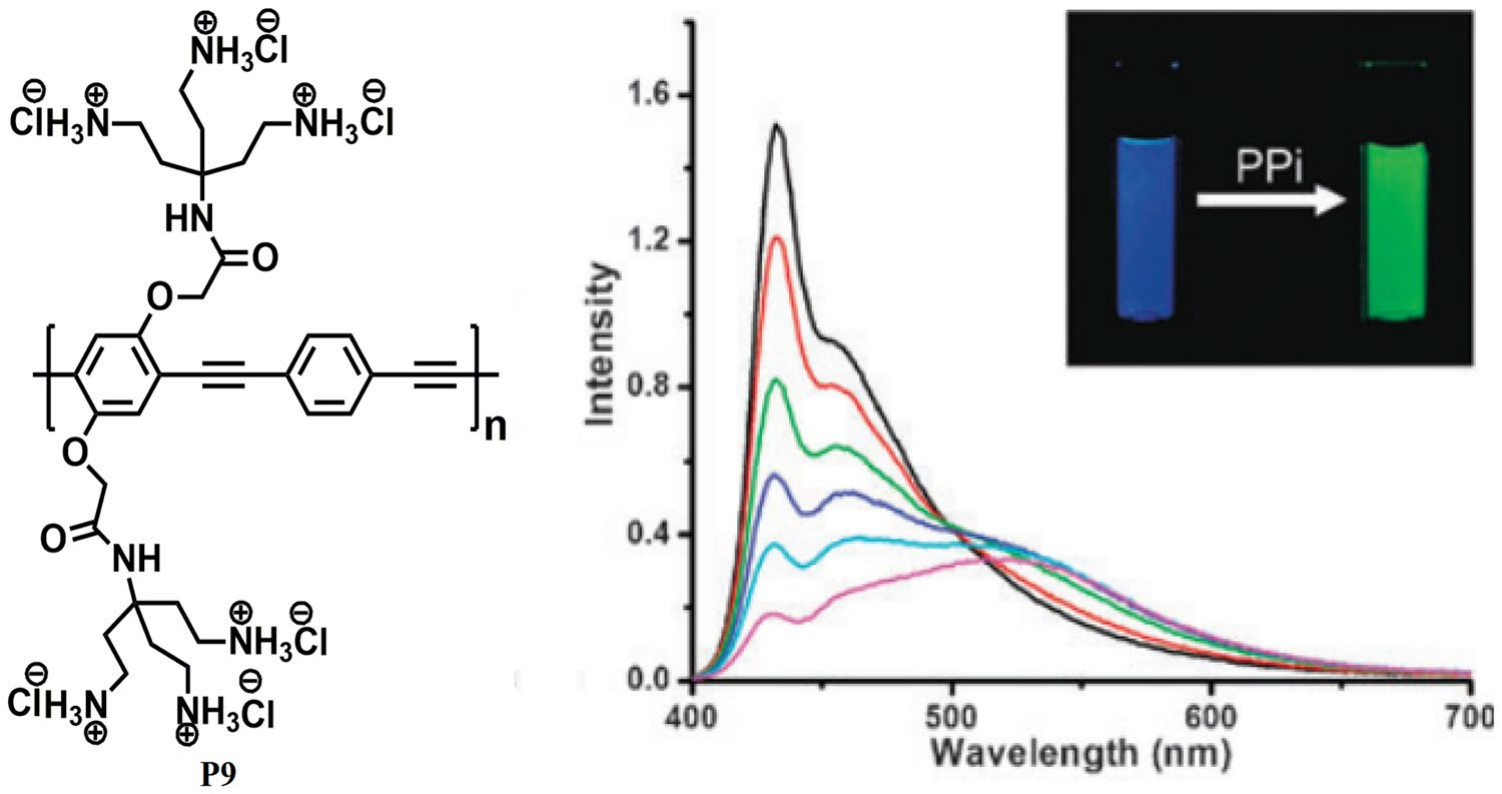
CP P9 developed for the naked-eye detection of PPi in aqueous solution. (The figure is reproduced with permission from Zhao and Schanze [47] *Copyright * *© 2010*, Royal Society Chemistry)

In 2010, Zhao and Schanze [47] developed a cationic CPE **P9** (Fig. 10) based on poly (phenylene ethynylene) via Sonogashira coupling reaction with polyamine side chains and explored its application in the ratiometric detection of pyrophosphate (PPi) with a detection limit of 340 nM. Polymer **P9** was designed in such a way that it shows redshifts in both emission and absorption spectra upon aggregation. This phenomenon of colour change by switching from the free chain state to the aggregate state was then explored for the naked-eye detection of PPi in an aqueous solution via analyte-induced aggregation of CPE. Upon gradual addition of increasing concentration of PPi into the aqueous solution of **P9**, the absorption band at 400 nm decreases and a new absorption band appear at 430 nm. The emission spectra also reveal the clear transition between the two states as the blue emission with well-resolved peaks between 433–455 nm decreases in intensity with increasing PPi concentration accompanied by an increase in the green emission band at 520 nm. This large red shift of about 90 nm is due to the efficient intermolecular exciton coupling among the polymer chains which results in a lowering of energy in the aggregate state.

**Fig. 11:**
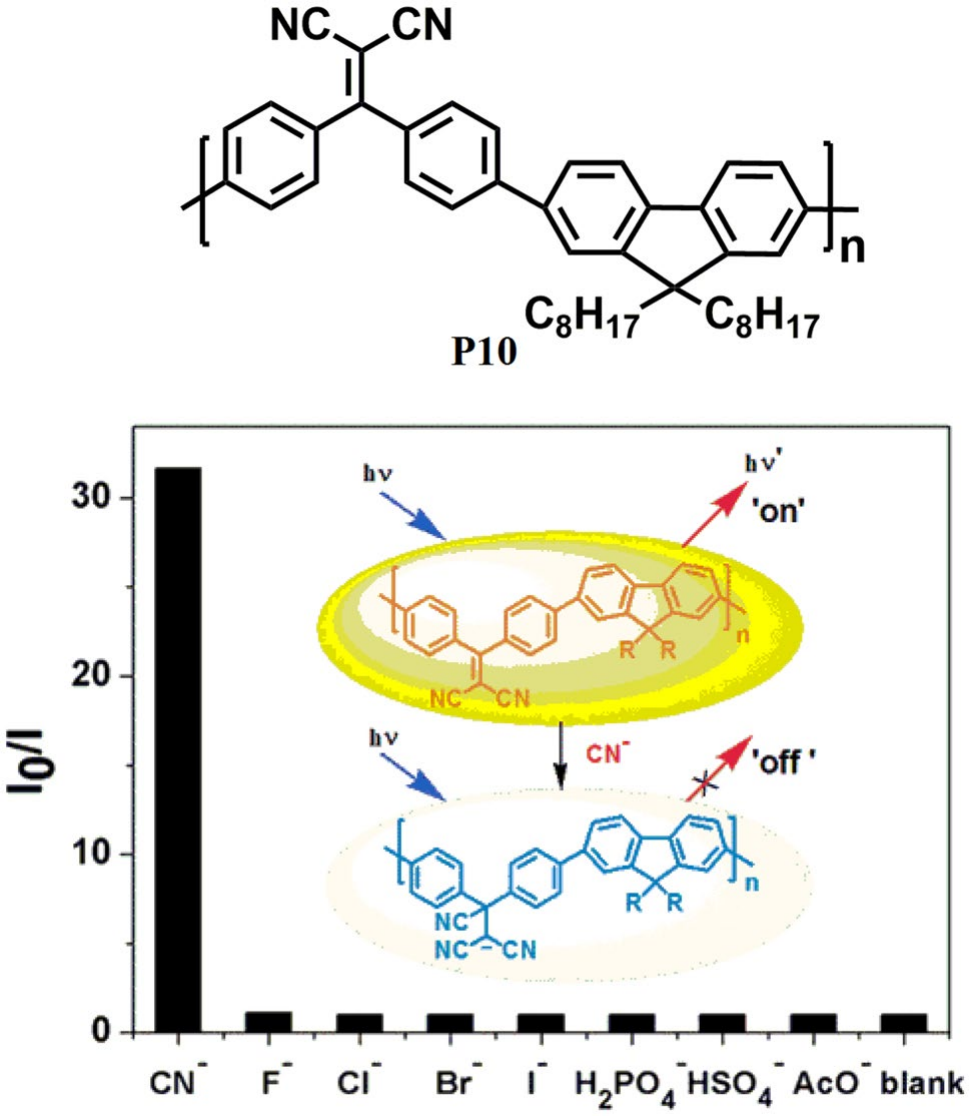
Detection of cyanide ion with a detection limit of 14 ppb using CP P10. (The figure is reproduced with permission from Wu et al. [48] Copyright © 2011, American Chemical Society)

In 2011, Wu et al. [48] developed a new CP **P10** (Fig. 11) based on polyfluorene and dicyano-vinyl as a highly selective and sensitive sensor for cyanide. The design of **P10** is based on the fact that the dicyano-vinyl group is a reactive site for cyanide via nucleophilic addition reaction which may disrupt the effective conjugation length of the polymer backbone and perturb its optical properties. Moreover, because of the exciton-diffusing property of CPs along its backbone, the nucleophilic adduct thus formed acts as a charge trapping site which may enhance the fluorescence quenching effect and ultimately result in the higher sensitivity of the CP for cyanide ion with a detection limit of 14 ppb.

**Fig. 12:**
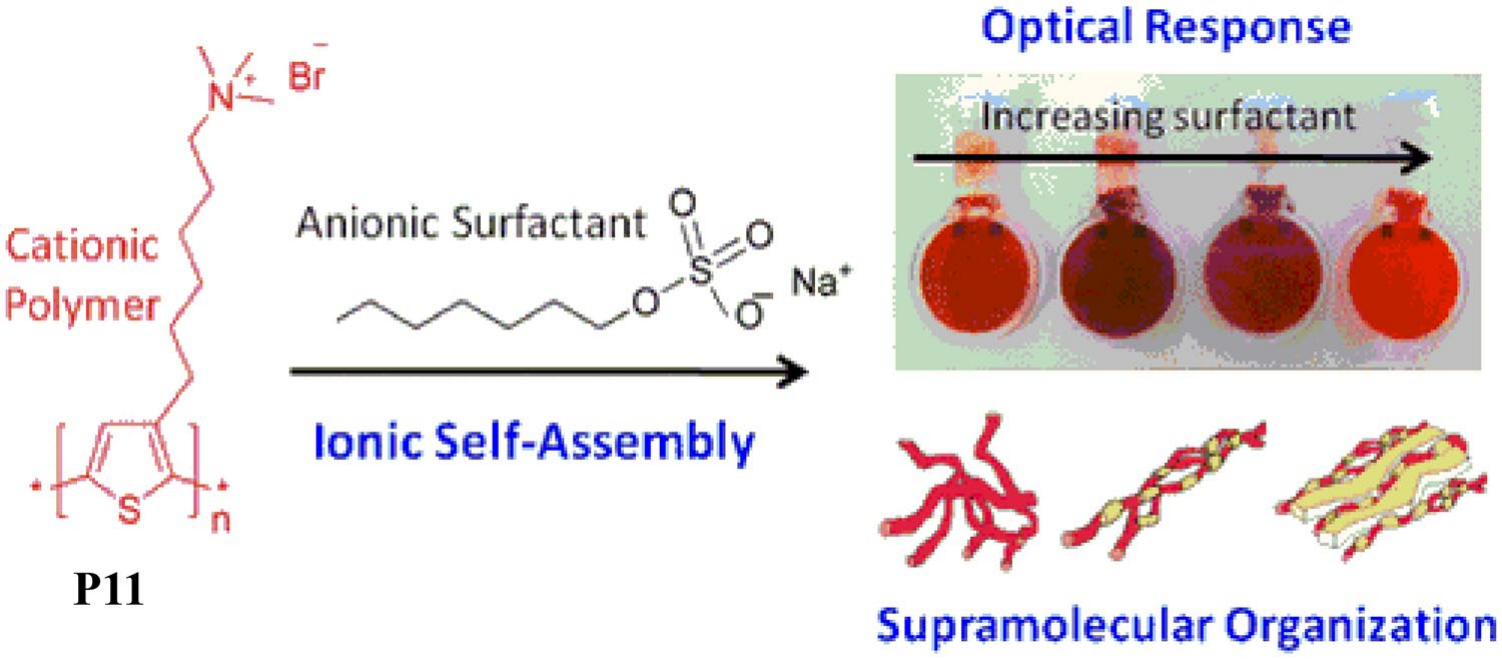
Structure P11 used for the detection of anionic surfactant. (The figure is reproduced with permission from Evans et al. [49] *Copyright * *© 2012*, American Chemical Society)

Furthermore, naked-eye detection of cyanide was also observed upon adding cyanide ions into the polymeric solution as the colour changes from yellow-green to colourless, while no colour change could be observed in presence of other common anions. In 2012, Evans et al. [49] studied the self-assembly complex formation between the cationic CPE **P11** (Fig. 12) and two anionic surfactants i.e., sodium octyl sulphate (SOS) and potassium heptadecafluoro-1-octanesulfonate (PFOS) both of which are industrially important. The absorption and emission spectra of **P11** were vividly changed in presence of anionic surfactants due to the formation of self-assembly via ionic complex formation. It was observed that subtle structural differences between these surfactants have helped to understand the effect of head group charge density, chain rigidity, and hydrophobicity on both the complex structure and optical properties using UV/vis absorption, fluorescence and Small-Angle Neutron Scattering (SANS). It was concluded that **P11** is capable of identifying anionic surfactants with distinct subgroups via dual-mode detection.

In 2015, Hussain et al. [50] synthesized a new CPE **P7** (Fig. 8a) via oxidative polymerization which displayed high selectivity and sensitivity towards most common anionic surfactants sodium dodecyl sulphate (SDS) and sodium dodecyl benzenesulfonate (SDBS) under various harsh conditions such as full pH range (1–14), urine, and in brine as well as in seawater. Polymer **P7** is very efficient in detecting and discriminating these moderately dissimilar anionic surfactants under acidic and basic conditions with a detection limit of 31.7 and 17.3 ppb respectively for SDBS and SDS in aqueous solution. Furthermore, **P7** could also remove these hazardous surfactants at very low levels from the water and other biological media such as urine in the form of gel or precipitate as a result of its differential aggregation behaviour due to inter-polymer cofacial arrangement via columbic attraction.

**Fig. 13:**
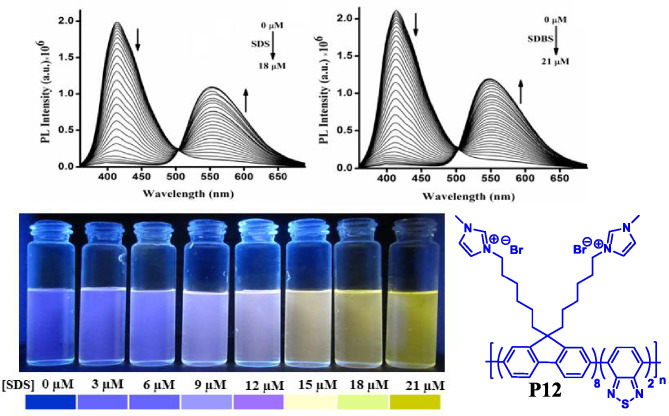
Ratiometric sensor for the detection of anionic surfactants SDS and SDBS using CP P12. (The figure is reproduced with permission from Malik et al. [51] *Copyright * *© 2016*, American Chemical Society)

In the year 2016, Malik et al. [51] developed **P12** a ratiometric sensor for the naked eye detection of anionic surfactants such as SDS and SDBS with a detection limit of 34 ppb and 45 ppb respectively in aqueous media. The polymer P12 (PFBT-MI) changed its colour from blue to yellowish green upon interaction with the anionic surfactants which is due to the inter-molecular FRET within the polymer chains driven by electrostatic interaction and hydrophilic interaction between the polymer and the surfactant (Fig. 13).

### Detection of biomolecules

**Fig. 14:**
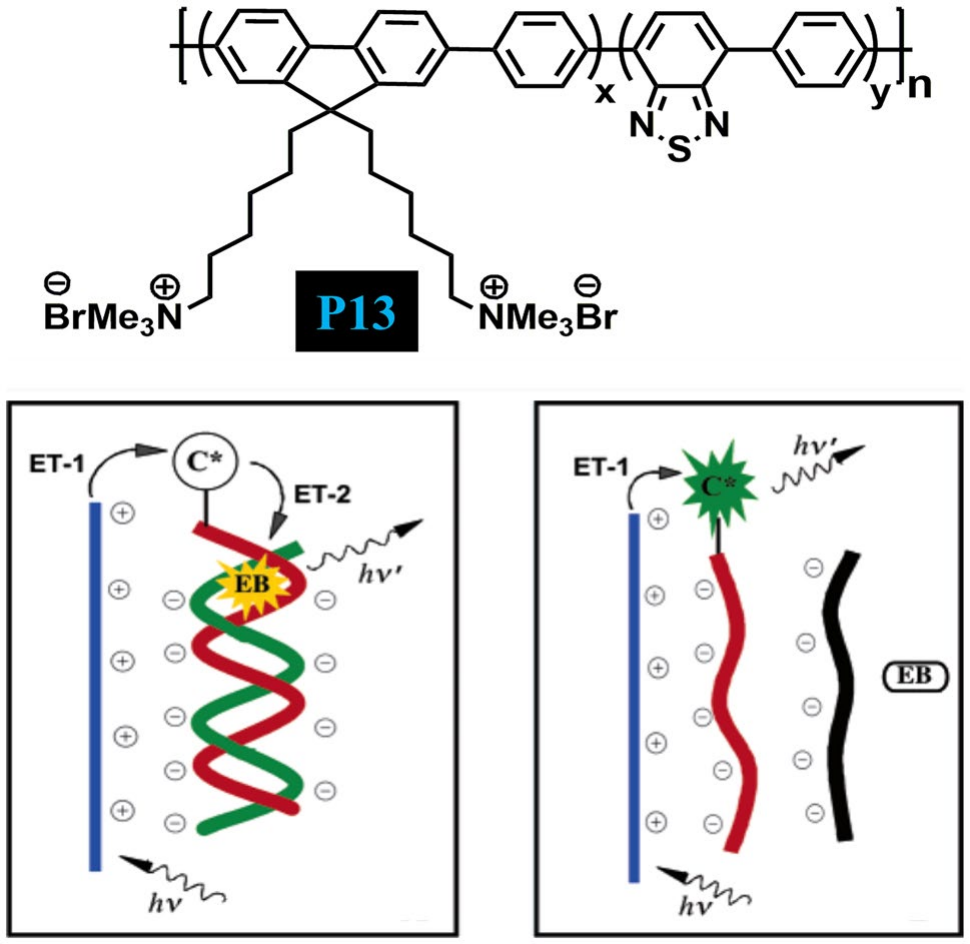
CP P13 enabled to determine DNA concentration vai the phenomena of FRET. (The figure is reproduced with permission from Liu and Bazan [52] *Copyright * *© 2004*, American Chemical Society)

In 2004, Liu and Bazan [52] synthesised a multipurpose cationic CPE **P13** (Fig. 14) via the Suzuki-coupling copolymerization technique by introducing 5% 2,1,3-benzothiadiazole (BT) units into cationic poly(fluorine-co-phenylene) to obtain two distinct emission colours from a single polymer chain. The cationic polymer **P13** strongly interacts with oppositely charged DNA molecules in an aqueous solution, resulting in the aggregation of polymer chains. Such inter-polymer interactions lead to an increase in the local concentration of benzothiadiazole (BT) units (responsible for FRET) and inter-chain contacts that subsequently improve electronic coupling between the optical partners. This ultimately facilitates the energy transfer process from the fluorine-phenylene (donor) segments to BT (acceptor) units, thus enabling the change in emission colour of the solution from blue to a green that aided in the determination of DNA concentration. Furthermore, the authors demonstrated that polymer **P13** could also be employed as a three-colour DNA assay using a PNA-C* strand. Depending upon the content of the solution, three different emission colours could be obtained i.e. (i) blue (in the absence of DNA), (ii) green (in the presence of non-complementary ssDNA) and (iii) red (in the presence of complementary ssDNA). Taking advantage of the signal amplification effect of CPs, fine-tuning of electrostatic and optical events leads to multicolour biosensing assay.

**Fig. 15:**
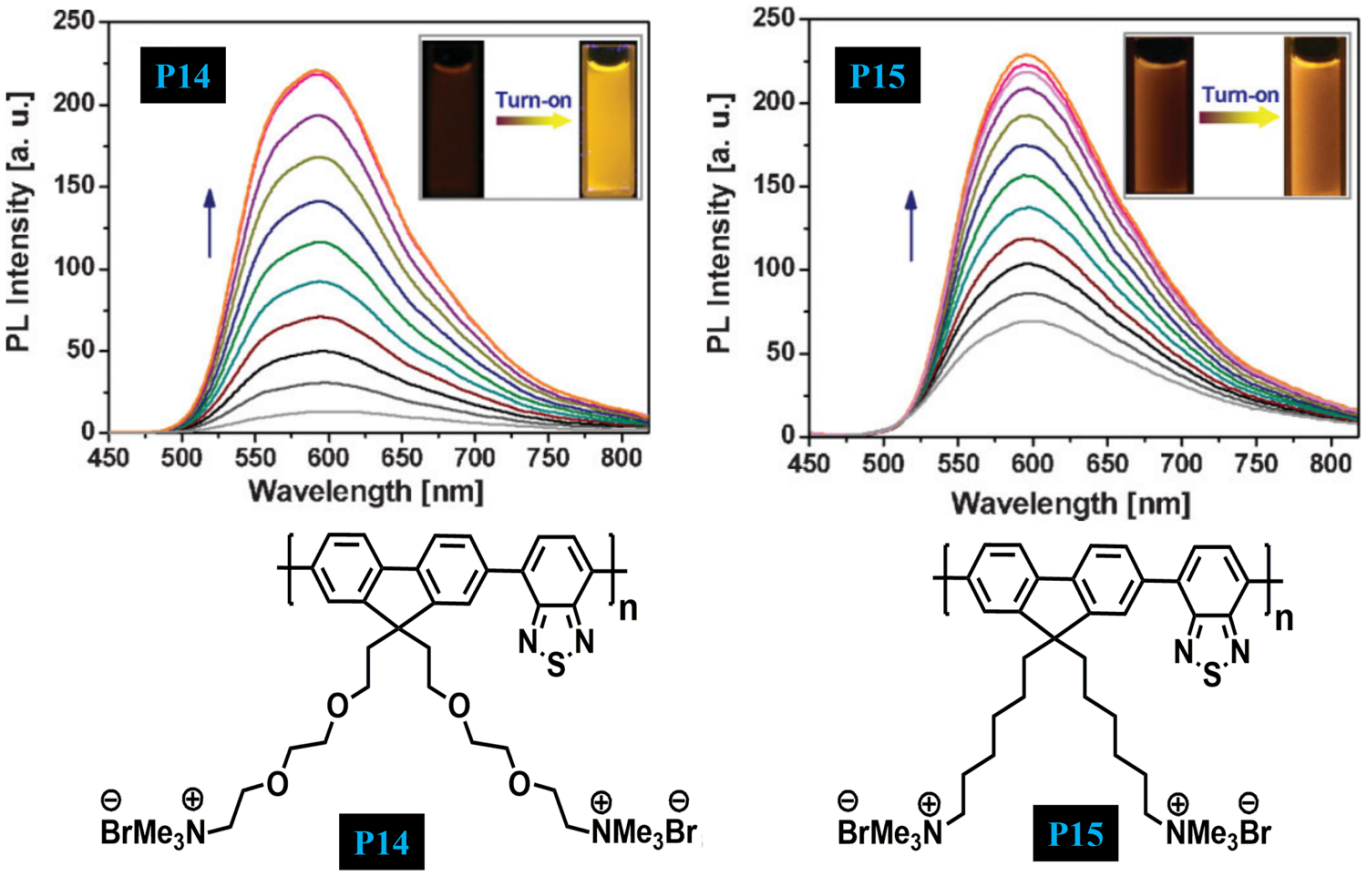
Turn On emission spectra of P14 and P15 for the quantification of heparin via complexation-induced aggregation. (The figure is reproduced with permission from Pu and Liu [53] *Copyright* © 2009 Wiley‐VCH GmbH)

In 2009, Pu and Liu [53] designed and synthesized two cationic CPEs **P14** and **P15** which differ only by side chains (Fig. 15). This benzothiadiazole (BT) based CPEs shows low fluorescence emission in an aqueous solution while as its fluorescence increases in the aggregated state. Taking the advantage of complexation-induced aggregation behaviour, a fluorescence turn-on sensor has been developed for the detection and quantification of heparin. A substantial increase in the fluorescence response of the polymer in presence of heparin as compared to its analogue hyaluronic acid (HA) is because of its strong complexation with heparin, thus allowing discrimination of heparin from HA. The advantage of this strategy is that it does not require a pre-quenching technique and the analyte can be detected directly using this polymer system.

**Fig. 16:**
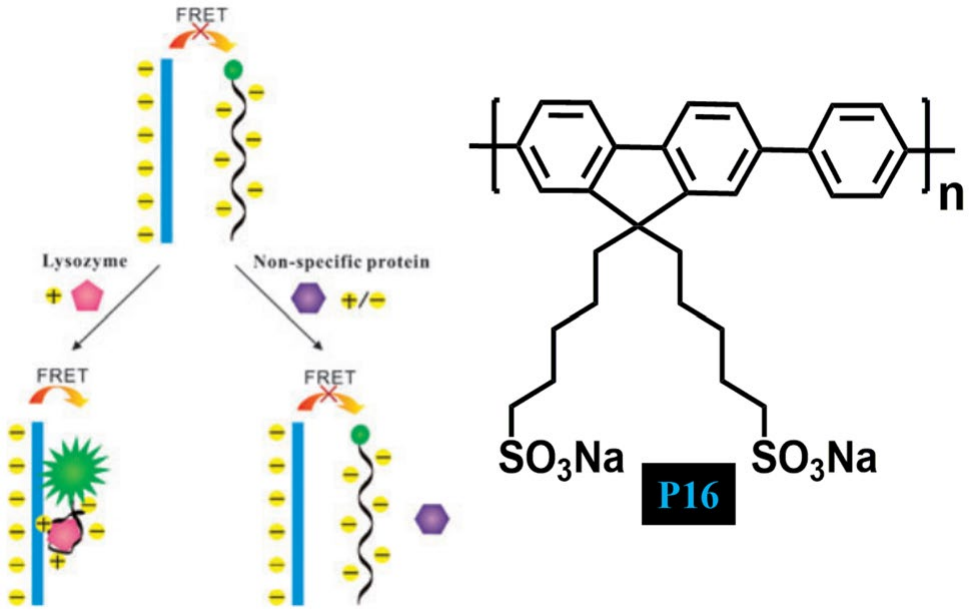
CP P16 has been explored for the development of new protein detection strategy via aptamer–target binding to trigger FRET from CP to labelled aptamer. (The figure is reproduced with permission from Wang and Liu [54] Copyright © 2009, Royal Society of Chemistry)

In 2009, Wang and Liu [54] designed a ligand-analyte binding facilitated energy transfer strategy for lysozyme detection using anionic CPE **P16** (Fig. 16) and dye-labelled aptamer. When the lysozyme interacts with the dye-labelled aptamer FRET occurs from **P16** to dye because of the strong electrostatic interactions and green colour formation. In the absence of lysozyme, no FRET happens and the colour of the solution is blue. The simplicity, sensitivity and specificity of this method have been explored by using lysozyme and lysozyme aptamer which has not been observed in other methods like electrochemistry, ELISA, and aptamer assay-based lysozyme assay therefore, this method has been successfully applied in biological media.

**Fig. 17:**
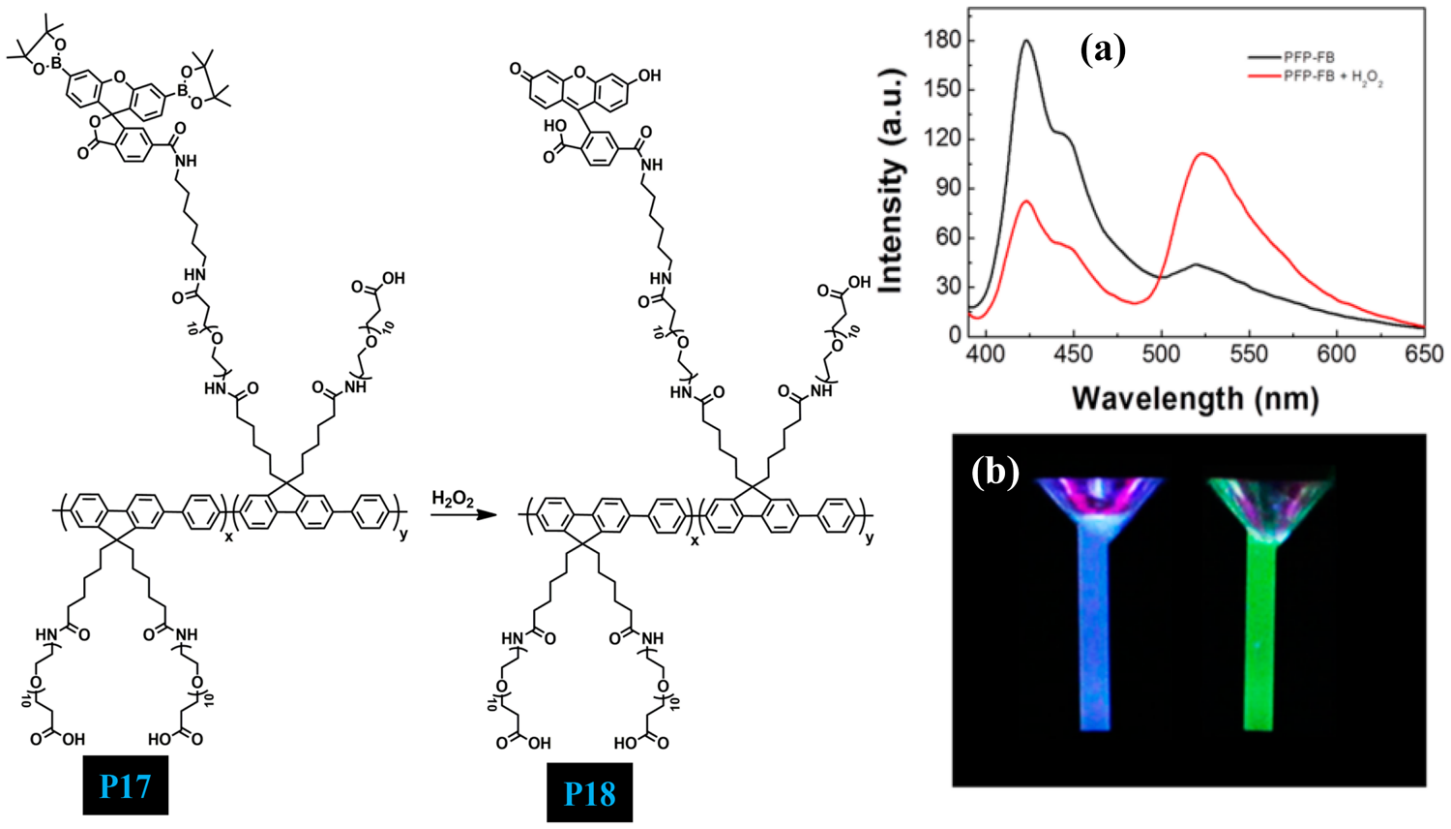
**a** PL emission spectra of P17 in the absence and presence of H_2_O_2_. **b** Photograph of P17 taken under UV in the absence and presence of H_2_O_2_. (The figure is reproduced with permission from Wang et al. [55] Copyright © 2015, American Chemical Society)

**Fig. 18:**
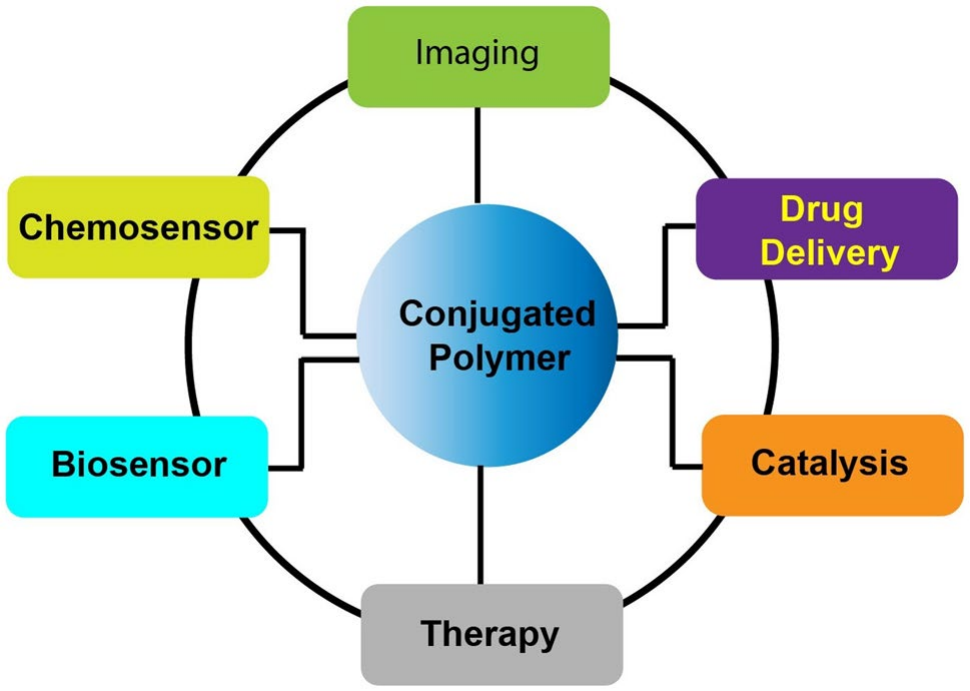
Table of content depicting applications of CPs

In 2015, Wang et al. [55] designed new water-soluble CP **P17** (Fig. 17) with a PEG side chain to which the carboxylic acid group and boronate-protected fluorescein are covalently attached. Carboxylic acid functionality and PEG chain not only increase the solubility of the polymer in water but also reduces nonspecific interactions with the cells and allow further modification of CP (Fig. 18). Efficient fluorescence resonance energy transfer (FRET) takes place from the **P18** backbone to the fluorescein unit in the presence of H_2_O_2_ upon excitation at 380 nm and the colour of the polymer solution changes from blue to green. Therefore, the polymer probe shows a good ratiometric fluorescence response to H_2_O_2_ that was generated from choline and acetylcholine (Ach) under AChE/ChOx enzyme-coupled reaction. The present strategy realizes choline and ACh detection in a simple and selective manner and presents high specificity to choline and ACh, respectively (Table 2).

**Table 2:** Various other Conjugated Polymers and their applications

**Conjugated** **Polymer**	**Excitation**	**Emission**	**Sensing Analyte**	**Sensing** **Mechanism**	**Ref**
**PFEP**	405 nm	525 nm	CD44	Disruption of FRET	[56]
**PFBT**	473 nm	540 nm	Glutathione S-transferase (GST)	Disruption of FRET	[57]
**PBEC**	430 nm	554 nm	Glutathione (GSH)	Disruption of FRET	[58]
**PFO**	380 nm	586 nm	Tyrosinase (TR)	Photo-induced electron transfer (PET)	[59]
**PFPy**	430 nm	553 nm	TNT	RET and FRET	[60]
**PFTBTCOOH**	380 nm	425 nm	Biogenic amines	Aggregation induced FRET	[61]
**PFBT**	370 nm	419& 530 nm	Picric acid	IFE	[62]
**PF-DBT-PEG**	380 nm	420 nm	Thiol	Aggregation induced FRET	[63]
**PF-DBTBIMEG**	380 nm	420 nm	Adenosine triphosphate (ATP)	Aggregation induced FRET	[64]
**PMI**	325 nm	406 nm	Nitro-explosive	RET	[44]
**PFPBA**	410 nm	529 nm	Dopamine	Charge transfer (CT)	[65]
**PMI**	325 nm	406 nm	Flavins	FRET	[66]
**PPE**	380 nm	500–580 nm	Imaging of mammalian cells	Electrostatic interaction	[67]
**PTPEDC**	350 nm	650 nm	in vitro cancer cell-ablation & in vivo zebrafish liver tumor treatment	Photodynamic	[68]
**PFMI**	370 nm	420 nm	Picric acid	RET	[45]
**PPE**	380 nm	500 nm	SARS-CoV-2	Photodynamic/electrostatic interaction	[69]

## Conclusions

In this article, recent progress in the design and synthesis of various CPs by exploring various coupling reactions has been summarized. The perspective includes a brief introduction about CPs, followed by a comprehensive study of the sensing mechanism involved. Hence, a new class of materials with exceptional photo-physical properties, good biocompatibility, low cytotoxicity, strong emission brightness and resistance to photobleaching have emerged which resulted in the development of highly selective and sensitive chemical and biological sensors, materials for efficient drug delivery, drug screening, cancer therapeutics, imaging, etc. In conclusion, there is a good potential and opportunity for CPs based approaches to be incorporated into multidisciplinary areas of chemical and biological sciences in the near future.
